# Population Genetics of *Plasmodium vivax* in the Peruvian Amazon

**DOI:** 10.1371/journal.pntd.0004376

**Published:** 2016-01-14

**Authors:** Christopher Delgado-Ratto, Dionicia Gamboa, Veronica E. Soto-Calle, Peter Van den Eede, Eliana Torres, Luis Sánchez-Martínez, Juan Contreras-Mancilla, Anna Rosanas-Urgell, Hugo Rodriguez Ferrucci, Alejandro Llanos-Cuentas, Annette Erhart, Jean-Pierre Van geertruyden, Umberto D’Alessandro

**Affiliations:** 1 Epidemiology for Global Health Institute, University of Antwerp, Antwerp, Belgium; 2 Institute of Tropical Medicine Alexander von Humboldt, Universidad Peruana Cayetano Heredia, Lima, Peru; 3 Departamento de Ciencias Celulares y Moleculares, Facultad de Ciencias y Filosofía, Universidad Peruana Cayetano Heredia, Lima, Peru; 4 Department of Biomedical Sciences, Institute of Tropical Medicine, Antwerp, Belgium; 5 Ministry of Health of Peru, Loreto, Peru; 6 Department of Public Health, Institute of Tropical Medicine, Antwerp, Belgium; 7 Medical Research Council Unit, Fajara, The Gambia; 8 London School of Hygiene and Tropical Medicine, London, United Kingdom; Walter and Eliza Hall Institute, AUSTRALIA

## Abstract

**Background:**

Characterizing the parasite dynamics and population structure provides useful information to understand the dynamic of transmission and to better target control interventions. Despite considerable efforts for its control, *vivax* malaria remains a major health problem in Peru. In this study, we have explored the population genetics of *Plasmodium vivax* isolates from Iquitos, the main city in the Peruvian Amazon, and 25 neighbouring peri-urban as well as rural villages along the Iquitos-Nauta Road.

**Methodology/ Results:**

From April to December 2008, 292 *P*. *vivax* isolates were collected and successfully genotyped using 14 neutral microsatellites. Analysis of the molecular data revealed a similar proportion of monoclonal and polyclonal infections in urban areas, while in rural areas monoclonal infections were predominant (*p* = 0.002). Multiplicity of infection was higher in urban (MOI = 1.5–2) compared to rural areas (MOI = 1) (*p* = 0.003). The level of genetic diversity was similar in all areas (*He* = 0.66–0.76, *p* = 0.32) though genetic differentiation between areas was substantial (*PHI*_PT_ = 0.17, p<0.0001). Principal coordinate analysis showed a marked differentiation between parasites from urban and rural areas. Linkage disequilibrium was detected in all the areas ($I_{A}^{s}$ = 0.08–0.49, for all *p*<0.0001). Gene flow among the areas was stablished through Bayesian analysis of migration models. Recent bottleneck events were detected in 4 areas and a recent parasite expansion in one of the isolated areas. In total, 87 unique haplotypes grouped in 2 or 3 genetic clusters described a sub-structured parasite population.

**Conclusion/Significance:**

Our study shows a sub-structured parasite population with clonal propagation, with most of its components recently affected by bottleneck events. Iquitos city is the main source of parasite spreading for all the peripheral study areas. The routes of transmission and gene flow and the reduction of the parasite population described are important from the public health perspective as well for the formulation of future control policies.

## Author Summary

We present the population genetics of malaria vivax parasites in a large area of the Peruvian Amazon. Our results showed that the parasite population had a predominant clonal propagation, reproducing themselves with identically or closely related parasites; therefore, the same genetic characteristics are maintained in the offspring. The clonal propagation may favour the higher levels of genetic differentiation among the parasites from isolated areas compared to areas where human migration is common. The patterns of gene flow have been established, finding Iquitos city as a reservoir of parasite genetic variability. Moreover, a recent reduction of the parasite population was observed in areas where recent control activities were performed. This research provides a picture of the nature and dynamics of the parasite population which have a significant impact in the malaria epidemiology; therefore, this knowledge is crucial for the development of efficient control policies.

## Introduction

According to the World Health Organization (WHO), *Plasmodium vivax* caused about 14.2 million malaria cases outside sub-Saharan Africa in 2013 [1]. Despite considerable efforts, Asian and South American countries are still far from achieving malaria elimination [2]. In Peru, the vast majority of malaria cases (76% of 64,673) was reported in the Amazon basin area (Loreto region) for 2014 and about 83% of them are due to *P*. *vivax* [3]. Many *P*. *vivax* infections are asymptomatic and undetectable by microscopy, providing a potentially important reservoir sustaining local transmission [4–7]. In addition, multiple infections recur even after the administration of the WHO-recommended radical cure treatment against blood- and hepatic-parasite stages (chloroquine and primaquine) [5, 6, 8]. To understand the epidemiology, distribution and transmission dynamics of *P*. *vivax* and thus improve its control, it is necessary to unravel the parasite population genetics and dynamics [9, 10]. Such information would be extremely useful for the monitoring and evaluation of control activities, both in the short and long term [10–12].

The extreme genetic variations in the *P*. *vivax* populations has already been reported from several endemic areas [10, 13]. In the Peruvian Amazon, the few observations available on *P*. *vivax* population genetics were collected in small areas (dispersed villages and communities) and reported heterogeneous and clonal parasite populations [6, 14–16]. Hereby, we report the genetic diversity and population genetics of the *P*. *vivax* parasite population from the most important urban city in the Peruvian Amazon and 25 villages located around and along the Iquitos-Nauta road.

## Methods

### Study sites

**Fig 1:**
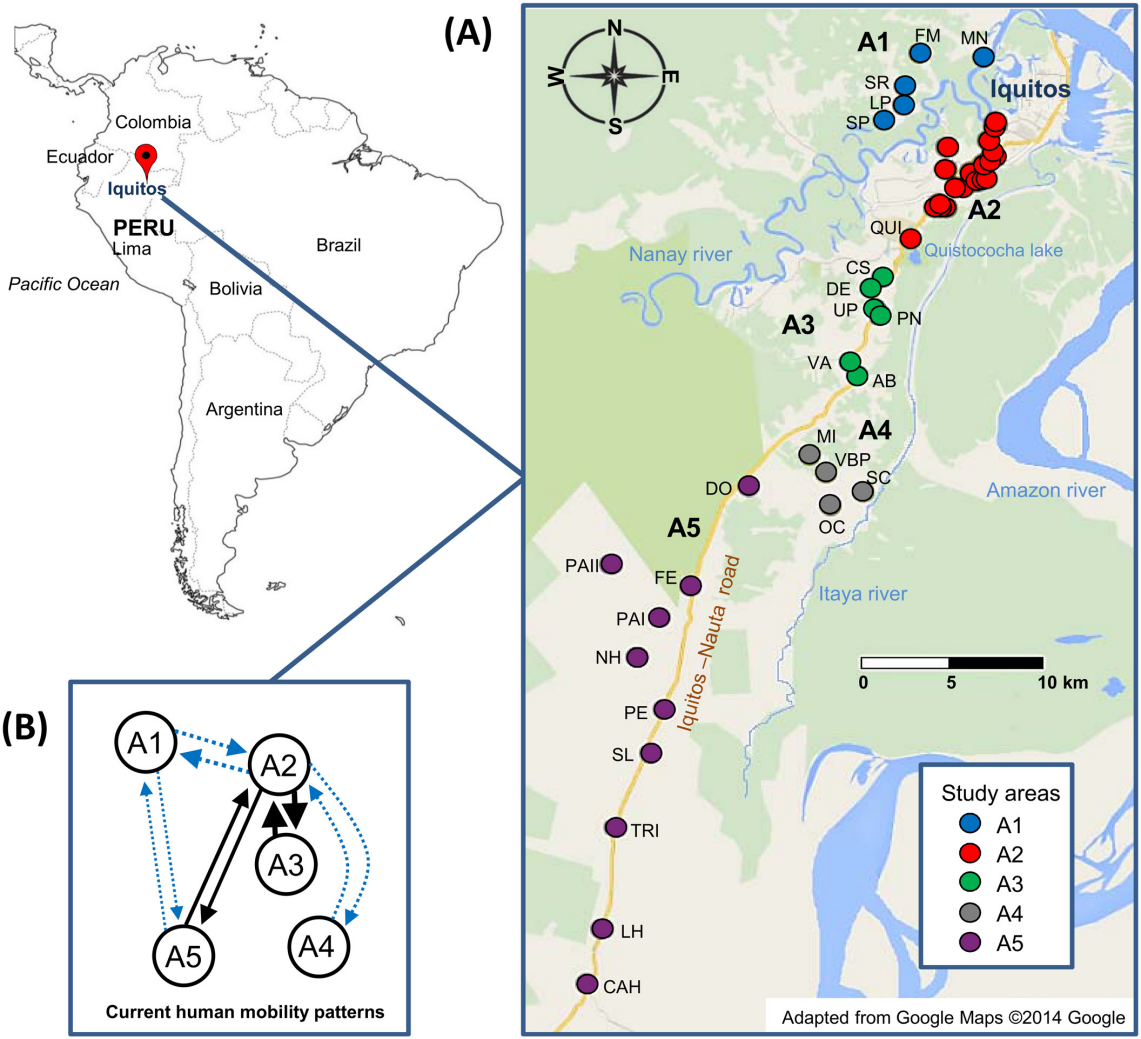
Study areas and human migration patterns in and around Iquitos city and along the Iquitos-Nauta road (Loreto, Peru). **(A)** Number of genotyped *P*. *vivax* isolates per area. Overall = 292: A1 = 105 (36.0%), A2 = 70 (24.0%), A3 = 32 (11.0%), A4 = 55 (18.8%) and A5 = 30 (10.3%). For detailed information of the villages that compose each area and frequency of isolates, see S1 Table. **(B)** The current human migration patterns are described by arrows indicating the direction and the occurrence of transit (thickness). Dotted blue arrows indicate that the transit involves crossing and/or navigating through a river while the solid black arrows indicate transit through the Iquitos Nauta road.

The clinical isolates were collected during an initial screening of the study sites in the Peruvian Amazon (April 2008) followed by active case detection of all fever cases (April to December 2008) during a longitudinal study assessing the efficacy of the recommended radical cure treatment for *P*. *vivax* malaria infection (chloroquine 25 mg/kg/day for 3 days and primaquine 0.5 mg//kg/day for 7 days) [17]. The study was conducted both within Iquitos city and in 25 neighbouring villages (Loreto region), some of them along the Iquitos-Nauta Road. Villages were geographically stratified in 5 study areas (A1- A5) (Fig 1A and S1 Table): A1 (3–7 km northwest from Iquitos city and only accessible by boat), A2 (Iquitos city and peripheral villages), A3 (villages situated along the Iquitos-Nauta Road, 11–13 km southwest from Iquitos city), A4 (villages situated 21 km southwest of Iquitos city and 2–9 km far from the Iquitos-Nauta Road) and A5 (villages situated 26–58 km southwest from Iquitos city, most of them along the Iquitos-Nauta road). Participants’ demographics are described in S1 Table.

In Fig 1B, we describe the main human mobility patterns in the study areas. Most human mobility occurs around A2 (Iquitos city, economic centre and big markets). Indeed, some people commute every day to A2 but live in A1 or A3, others travel from A5 to A2 during the weekends crossing the Nanay river or come from A4 a couple of times per year navigating along the Itaya river to sell their goods. Some sporadic movement between A1 and A5, crossing the Nanay river, is observed.

The study population consisted primarily of mestizos of low socioeconomic status. Villages located along Iquitos-Nauta road or next to the river had no electricity and in most cases drinking water was taken from the river or natural springs. Malaria transmission is perennial with peaks from November to May (rainy season)[18], and the majority of malaria cases are due to *P*. *vivax*. Recurrent sub-patent and asymptomatic infections are frequent [5, 6]. *Anopheles darlingi* is the main anthropophilic and exo/endophilic vector [19]. Malaria prevention and control activities were conducted before the sample collection in Loreto region including all our study areas but in A4 (S1 Table) as part of PAMAFRO (Malaria Control Program in Andean-country Border Regions) which started in 2005 [20].

### Sample collection

Patients were examined daily during the treatment and followed up weekly with blood sampling according to WHO guidelines for *P*. *vivax* drug efficacy during the first 28 days [WHO, 2009]; and systematic monthly follow-up was carried out thereafter. For the purpose of the current study, all D0 (before-) and D1 (after treatment) isolates were analysed, including a blood sample for microscopy (thick and thick film) and a blood spot on filter paper (BSFP) (Whatman grade 3, Whatman, Springfield Mill, USA).

### Ethics statement

The study was approved by the Ethical Boards of Universidad Peruana Cayetano Heredia, Peru (Project PVIVAX-UPCH, SIDISI code: 053256), the institutional review board of the Institute of Tropical Medicine Antwerp and the University Hospital of Antwerp, Belgium. Adult participants provided informed written consent, and a parent or guardian of any child participant provided informed written consent on their behalf.

### Laboratory methods

All slides were read by microscopy (thin and thick smear) to confirm *P*. *vivax* infection and estimate the parasite density (number of asexual parasites for 200 white blood cells (WBC) assuming 8000 WBC/μl) [6, 21]. Parasite and human DNA was extracted using the Chelex method [22]. *P*. *vivax* mono-infections were confirmed by species-specific PCR (ssPCR) [23] and genotyped using a panel of 14 well-described microsatellite markers (MS), namely MS1, MS2, MS3, MS4, MS5, MS6, MS7, MS8, MS9, MS10, MS12, MS15, MS16 and MS20 [24, 25]. Briefly, the DNA extracted from each sample was used to perform a separate PCR for each MS. The forward primer of each MS was labelled with a fluorophore to identify the size of the amplicon through capillary electrophoresis in a 3730 XL ABI sequencer (Applied Biosystems, Foster City, CA, USA) [6]. MS PCR was repeated for those isolates with no MS PCR amplification. Some isolates from A1 and A4 were previously analysed and already published [6, 14].

### Data analysis

The allele fragment sizes recovered from the capillary electrophoresis were determined using Genemapper (Applied Biosystems, Foster City, CA, USA). Only fragments with ≥100 relative fluorescence units (RFU) were considered as ‘real’ alleles. In case of the presence of two or more alleles, only alleles with RFU ≥30% of the dominant allele RFU were considered for further analysis. The genetic and statistical analysis were performed mainly at study areas level but whenever feasible also at the village level to assess the influence of individual villages on the areas. SPSS for Windows v.20 (IBM Corp., NY) was used to perform non-genetic statistical analysis.

#### Multiplicity of infection

Infections were classified as monoclonal (1 allele per loci) or polyclonal (≥2 alleles in at least one locus). The haplotypes (unique combination of 14-MS alleles) were determined using GenAlEx v.6.5 [26, 27]. The predominant haplotypes were recovered from polyclonal infections based on the highest peak of the alleles [28, 29]. The multiplicity of infection (MOI, minimal number of distinct “clones” or haplotypes within a isolate) was estimated by taking the locus with the highest number of alleles as a proxy [6]. The proportions of polyclonal infections were compared between areas with Pearson χ^2^ test and the average MOI compared using the Kruskal-Wallis test.

#### Genetic diversity and parasite differentiation

The mean number of alleles and the number of private alleles (alleles unique to a single area) were determined for each area using GenAlEx. The allelic richness, a non-biased-by-sample size measure of the number of alleles, was determined using FSTAT v.2.9.3 [30]. The expected heterozygosity (*He*) was determined per locus and per study area using GenAlEx. The *He* represents the probability of finding a different allele for a given locus in any pair of samples randomly drawn from the same population. *He* varies from no diversity at all (0) to highly diverse (1). Overall monoclonal/polyclonal infections with complete/incomplete MS allele data were included for the calculations of these metrics. The *He* and the allelic richness were compared among study areas using Kruskal-Wallis Test.

The 2-level hierarchical genetic variation of the parasites between and within study areas was analysed by the Analysis of Molecular Variance (AMOVA) using GenAlEx [26, 27], and the *PHI*_PT_ statistic, an estimate of the proportion of the parasites’ genetic variance between areas relative to the total variance, was calculated. When a third hierarchical level (villages) was included the proportion of variance between the areas (*PHI*_RT_), among the villages (*PHI*_PR_) and within the villages (*PHI*_PT_) were calculated. Probabilities for the AMOVA indexes were calculated based on individual randomizations (9999 permutations) [26, 27]. Pairwise comparisons of the genetic differentiation between areas or villages were further evaluated calculating the pairwise *PHI*_PT_ values. *PHI*_PT_, analogue of the fixation index *Fst*, suppresses the within-population variance and ranges from 0 (no differentiation) to 1 (full differentiation). *PHI*_PT_ was determined only for isolates with full haplotype data [26, 27].

Principal Coordinate Analysis (PCoA), which plots the major relationships within a multivariate dataset, was generated with GenAlEx by intercomparison of geographic areas and villages using the *PHI*_PT_ matrix obtained via AMOVA and the individual-by-individual binary genetic distance matrix (for later genetic relatedness analysis) [26, 27]. Only isolates with full allele data were included on the PCoA. Mantel test for Matrix Correspondence was performed using GenAlEx to test the occurrence of a positive correlation (*Rxy*>0) between the genetic *PHI*_PT_ matrix and geographic distances, so called the Isolation-by-distance (IBD) hypothesis. Geographic distances were computed using geospatial data for each village and converting UTM to km with GenAlEx [26, 27].

#### Linkage disequilibrium

The presence of linkage disequilibrium (LD), the non-random association of alleles from different loci, was evaluated by determining the standardized Index of Association ($I_{A}^{s}$) as a measure of multilocus linkage using LIAN [31]. Bias due to equivocal assignment of haplotypes in polyclonal infections was assessed by determining the linkage in (i) both monoclonal and polyclonal infections, and (ii) in monoclonal infections only [32]. LD was also assessed on infections with unique haplotypes in order to differentiate between clonal propagation and epidemic expansion [29, 33]. Pairwise-loci LD was evaluated using FSTAT to discard physical linkage between loci located within the same contig (MS4-MS5, MS7-MS8 and MS12-MS15) [30].

Genetic structure. The genetic structure was explored using three different approaches using eBURST, STRUCTURE and GENODIVE. The software eBURST v.3 assigned the haplotypes to genetic clusters (‘*haplogroups*’) of parasites that shared the same alleles in at least 7–13 loci [34]. Haplotypes not complying with the clustering criteria were defined as singletons. The software STRUCTURE v.2.3.3 was used to elucidate the genetic structure and the most likely number of clusters (*K*)[35]. STRUCTURE runs were performed exploring *K* from 1 to 15 (10 iterations each), consisting of a burn-in period of 50,000 iterations followed by 150,000 Markov Chain Monte Carlo (MCMC) iterations, assuming a mixture model and correlated allele frequencies. The most likely *K* was defined according to Evanno *et al*.[35] by calculating the rate of change of *K*, Δ*K*, using STRUCTURE HARVESTER v0.6.94 [36]. The haplotypes with >85% ancestry were assigned to a specific cluster for later cluster differentiation analysis. Hierarchical structure were analysed within the identified clusters using sampling location as priors (LOCPRIOR option) and increasing the up to 20 iterations for each *K*, burn-in 50,000 iteration followed by 500,000 MCMC iterations [37]. Additional data parsing and formatting of the STRUCTURE results were subsequently done using CLUMMP [38] and DISTRUCT [39]. CLUMMP aligns the cluster assignment across replicate analyses while DISTRUCT performs a graphical display of the aligned cluster assignments. Genetic differentiation among the predicted genetic clusters was evaluated through AMOVA and PCoA. In order to confirm the genetic clustering of the parasites, an AMOVA-based *K*-means clustering method implemented in the software GENODIVE v.2.0b23 (OS X 10.6 operating system) was used [40]. This method combines the *K*-means clustering which divides a number of individuals into an *a priori* assigned number of groups (*K*) in such a way that the within-group diversity is minimized and the between-group is maximized [41]. In order to determine the optimal clustering, the *pseudo-F* statistic (the highest value = optimal clustering) was calculated using the simulated annealing with 150,000 steps as convergence method and the number of algorithm repeats set to 50. GENODIVE retrieves the *Rho* index (analogue of *Fst* ranging from 0 to 1) as a measure of genetic differentiation between the genetic clusters [40]. Only isolates with full haplotype data were included for eBURST, STRUCTURE and GENODIVE analyses, as well as isolates with up to 3 loci with missing data for the two last analyses. In order to verify the presence of independent genetic clusters, *He*, pairwise *PHI*_PT_ and multilocus and pairwise-loci LD were assessed as described above.

#### Network and gene glow analysis

PHYLOViZ v1.0 was used to display the phylogenetic relationship through minimum spanning trees (MST) among *P*. *vivax* haplotypes [42]. MST’s were constructed to explore the haplotypes’ genetic relationship in function to its geographic origin (areas differentially coloured) and to check the genetic clustering retrieved from STRUCTURE (clusters differentially coloured).

The gene flow was inferred through the assessment of 13 migration models using a Bayesian approach (MCMC analysis) based on the coalescence theory and implemented in MIGRATE-N v3.6 [43]. MIGRATE-N estimates the posterior probability distribution of the migration parameters: mutation-scaled migration rates (*M*) and population sizes (Θ); and calculates marginal log-likelihoods (log mL) for each model using a thermodynamic integration. The log mL’s were used to calculate the log Bayes factors (LBF, mL ratios) and probabilities [44]. The best model is the one with the highest log mL and the model ranking is made by comparison of the LBF’s and probabilities with respect to the best model. The models were built considering the genetic structuring gathered on the previous steps and on the knowledge on the people’s current mobility patterns in and around Iquitos (Fig 1B). Each migration model was conducted in MIGRATE-N deriving the starting migration parameters from *Fst*-like calculations and through a long chain of 1x10^6^ recorded genealogies at a sampling increment of 100 iterations after discarding the first 10,000 as burn-in for each locus. To improve the MCMC searches, static heating scheme with 4 chains with temperatures 1, 1.5, 3 and 1’000,000 was set. Uniform-distributed priors for the migration parameters were set to range 0–100. Brownian motion approximation to the ladder model was selected as microsatellite data model for faster calculations [45]. The number of immigrants per generation (*Nm*) was calculated as the product of *M* by Θ_(recipient)_. The effective population size (*Ne*) was also calculated: *Ne* = Θ/2*μ*; where *μ* = mutation rate per generation per locus, *μ* only described for *P*. *falciparum* = 1.59 x 10^−4^ (CI 95% 3.7x10^-4^–6.98x10^-5^) [46].

#### Bottleneck analysis

We tested the deviation from the mutation-drift equilibrium due to recent bottleneck or rapid expansion events in the parasite population using the software BOTTLENECK v1.2.02 [47, 48]. After a recent reduction of the effective population size, the extent of the reduction of the number of alleles (rare alleles mainly) would be higher compared to the reduction of genetic diversity (*He*). In contrast, the deficiency of *He* may indicate a recent population expansion. Using the Wilcoxon rank test, BOTTLENECK determines if a significant number of microsatellites exhibits excess or deficiency of *He* under the most strict mutational models: Infinite Allele Model (IAM) and the Stepwise Mutational Model (SMM) and also the Two-phase model (TPM) which allows to fractionate the proportion of mutational models [47, 49]. The test was performed initially grouping the isolates by their geographic origin using only 10 polymorphic microsatellites (MS1, MS3, MS5 and MS7 were not used for this analysis, average *He*<0.5). Since the microsatellites we used may not follow the same mutational model we also performed the analysis separately for perfect 3-4-bp repeats microsatellites (MS4, MS9, MS15, MS20) and imperfect microsatellites (MS2, MS6, MS8, MS10, MS12, MS16) which may tend to follow the SSM and IAM, respectively [47, 50]. The mutational models were simulated by 10,000 replications, and specifically for TPM the proportion of SSM was fractionated in 5–95% a variance among multiple steps of 30.

## Results

### Multiplicity of infection

**Fig 2:**
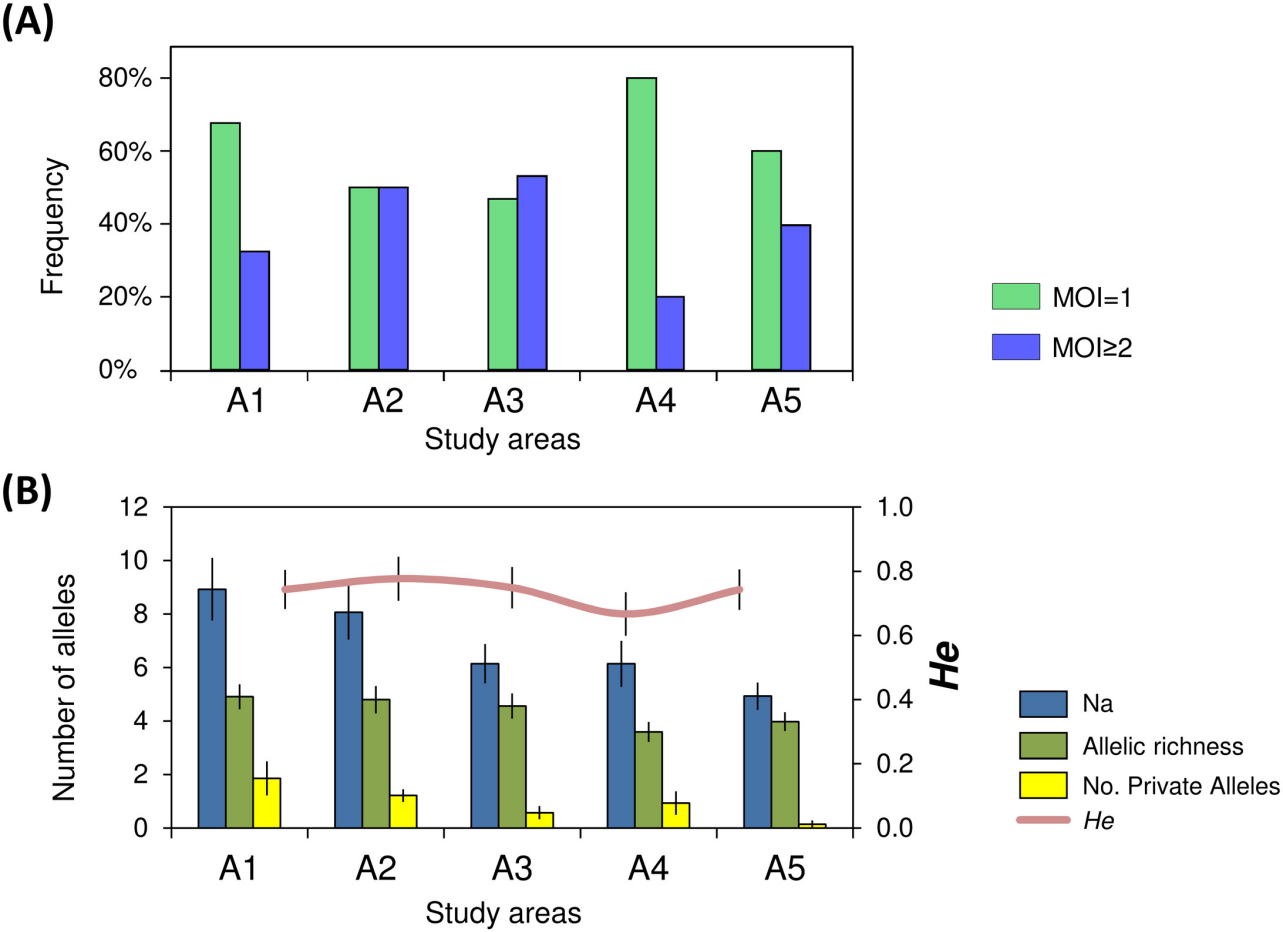
Distribution of monoclonal and polyclonal infections and allelic patterns between the study areas. All samples were considered for the analysis (A1 = 105, A2 = 70, A3 = 32, A4 = 55, A5 = 30). **(A)** Frequency of monoclonal (MOI = 1) and polyclonal infections (MOI≥2) per area. **(B)** Allelic patterns per area: Na = No. of different alleles, No. Private alleles = Number of alleles unique to a single area, He = Expected heterozygosity.

Overall we have analysed 292 *P*. *vivax* isolates (out of 302) with genotyping success on 56.2% (out of 292 isolates) with all 14 loci, 62.3% with 11–13 loci and 81.5% with 10 or less loci (efficiency of PCR amplification for each locus tabulated in S2 Table). Most of the isolates, 62.7% (183/292) were monoclonal infections and the polyclonal infections carried a minimum of 2 or 3 different haplotypes (34.6% and 2.7%, respectively). The proportion of mono/polyclonal infections significantly differed between areas (*p* = 0.002), i.e. A1 and A4 presented higher frequency of monoclonal infections while in A2 and A3 the proportions of mono/polyclonal infections were similar (Fig 2A). The median MOI was higher in A2 (MOI = 1.5) and A3 (MOI = 2) compared to the other areas (MOI = 1) (*p* = 0.003).

### Genetic diversity and parasite differentiation

The overall genetic diversity estimates among the areas described a median *He* = 0.74 (range 0.66–0.76), median allelic richness = 4.6 alleles (range 3.6–4.9), and did not differ significantly between areas (*He*: *p* = 0.32); allelic richness: *p* = 0.17), Fig 2B). Private alleles were less in A3 and almost absent in A5 than in the other areas (Fig 2B). The level of polymorphism (*He*) of each locus is presented in S2 Table. The AMOVA revealed that most of the genetic variation of the parasite population relied within areas (83%) (Table 1), though genetic differentiation between areas was also observed (*PHI*_PT_ = 0.17, *p* = 0.0001).

**Table 1:** Summary of hierarchical AMOVA for different geographical groupings and genetic clustering.

Grouping		Source of variation	df	Sum of squares	Estimated variation (%)	Statistic	*p*-value
**Geographic locations**	**By areas**^1^	Between areas	4	93.9	17%	*PHI*_PT_ = 0.17	0.0001
		Within areas	123	525.9	83%		
	**By villages**^2^	Between areas	4	136.8	22%	*PHI*_RT_ = 0.22	0.0002
		Between villages within areas	7	43.6	4%	*PHI*_PR_ = 0.06	0.0036
		Within villages	116	482.3	73%	*PHI*_PT_ = 0.27	0.0001
**Genetic clusters**^3^	**K = 2**	Between clusters	1	73	37%	*PHI*_PT_ = 0.37	0.0001
		Within clusters	87	355.3	63%		
	K = 3*	Between clusters	1	53.3	26%	*PHI*_PT_ = 0.26	0.0001
		Within clusters	79	338.7	74%		
	**K = 7**	Between clusters	6	224.9	59%	*PHI*_PT_ = 0.59	0.0001
		Within clusters	80	167.2	41%		

At 2-level hierarchical AMOVA: *PHI*_PT_ = estimate of the proportion of the parasites’ genetic variance between areas/clusters relative to the total variance. At 3-level hierarchical AMOVA: *PHI*_RT_, *PHI*_PR_ and *PHI*_PT_ = proportion of variance between the areas, between villages and within villages, respectively.

^1^Number of isolates: A1 (n = 60), A2 (n = 16), A3 (n = 10), A4 (n = 38), A5 (n = 4)

^2^Number of isolates: A1 (MN = 20, FM = 7, SR = 20, LP = 7, SP = 6), A2 (VS = 4, others A2 = 12), A3 (VA = 7, others A3 = 3), A4 (SC = 36, VBP = 2), A5 = 4.

^3^Genetic clustering assignment performed using STRUCTURE. Parasites classified as admixed were not considered for AMOVA. When *K* = 2 the clusters contained: 71 and 18 isolates; when *K* = 3: 58 and 23 isolates; and when *K* = 7: 23, 18, 15, 11, 10, 7 and 3 isolates.

* For *K* = 3, only two clusters were considered since no isolate was assigned as full member of the third cluster.

**Fig 3:**
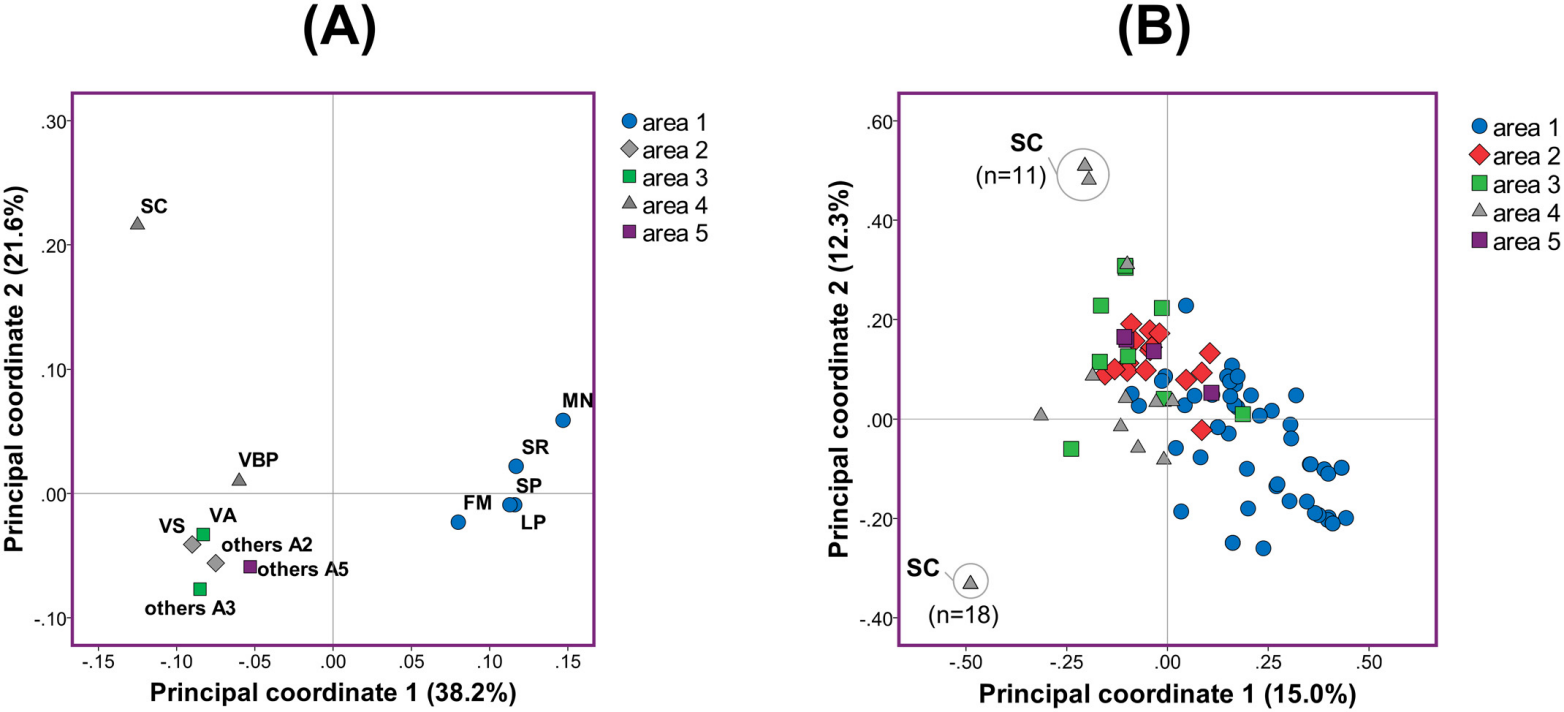
PCoA of the parasite population at village and individual haplotype level. (A) Principal coordinate analysis of the study areas resulted from intercomparison of individual villages. Percentages in the parenthesis indicate the proportion of total variation explained by each principal coordinate. The term “others…” was used to group villages within specific areas that had less than 2 isolates with known haplotype. (B) PCoA among the individual haplotypes within the areas. Areas are defined in Fig 1A and village abbreviations are detailed in S1 Table.

Pairwise calculations of the genetic differentiation and PCoA between areas and/or villages showed that parasites in the geographically isolated areas A1 and A4 were differentiated compared to those circulating in the other three areas with direct access to the Iquitos-Nauta road (Table 2 and Fig 3A). The first two coordinates of the PCoA explained 59.8% of the total variance pointing out clustering of parasites in villages from A1 (median *PHI*_PT_ 0.02 within A1), presence of related parasites in A2, A3 and A5 and genetic differentiation of parasites from San Carlos village (A4) (Fig 3A).

**Table 2:** *PHI*_PT_ values obtained from pairwise comparison between study areas (A1–A5) and between villages within each area.

		A1						A2			A3			A4		
		A1	MN	FM	SR	LP	SP	A2	VS	others A2	A3	VA	others A3	A4	SC	VBP
	**A1**															
	FM		**0.07**													
	SR		0.03	0.01												
A1	LP		**0.08**	0	0.01											
	SP		0.05	0	0.02	0										
	**A2**	**0.11**														
A2	VS		**0.33**	**0.26**	**0.28**	**0.29**	**0.29**									
	others A2		**0.23**	**0.12**	**0.17**	**0.16**	**0.15**		**0.09**							
A3	**A3**	**0.14**						**0.06**								
	VA		**0.3**	**0.18**	**0.23**	**0.26**	**0.26**		**0.34**	**0.15**						
	others A3		**0.3**	**0.10**	**0.21**	**0.2**	**0.18**		0.26	**0.10**		0.10				
	**A4**	**0.23**						**0.18**			**0.19**					
A4	SC		**0.36**	**0.32**	**0.33**	**0.37**	**0.36**		**0.37**	**0.28**		**0.31**	**0.32**			
	VBP		**0.26**	**0.10**	**0.14**	**0.11**	**0.12**		**0.23**	**0.09**		**0.21**	**0.06**		**0.18**	
A5	**A5**	**0.09**	**0.26**	0.10	0.17	0.13	**0.10**	-0.02	0.25	0.05	0.03	0.17	0.06	**0.19**	0.29	0.04

*PHI*_PT_ values given in bold were significantly greater than 0 at the 5% significance level. Areas (A1, A2…) are defined in Fig 1A and villages abbreviations are detailed in S1 Table.

When considering parasite populations per village, most of the genetic variation actually lied within villages (73% of the total genetic variation) with a high differentiation among the parasites within the villages (*PHI*_PT_ = 0.27, *p* = 0.0001), while little differentiation was found between the villages (*PHI*_PR_ = 0.06, *p* = 0.004) (Table 1). The PCoA at the individual haplotype level confirmed genetic differentiation among parasites within the same areas even within the same village: i.e. two different groups of haplotypes in San Carlos village (Fig 3B). The genetic distance of the parasite population was not correlated with geographic distance between villages (using *PHI*_PT_ matrix *Rxy* = -0.41 *p* = 0.17) (scatterplot of the genetic and geographic distances in S1 Fig).

### Linkage disequilibrium

**Fig 4:**
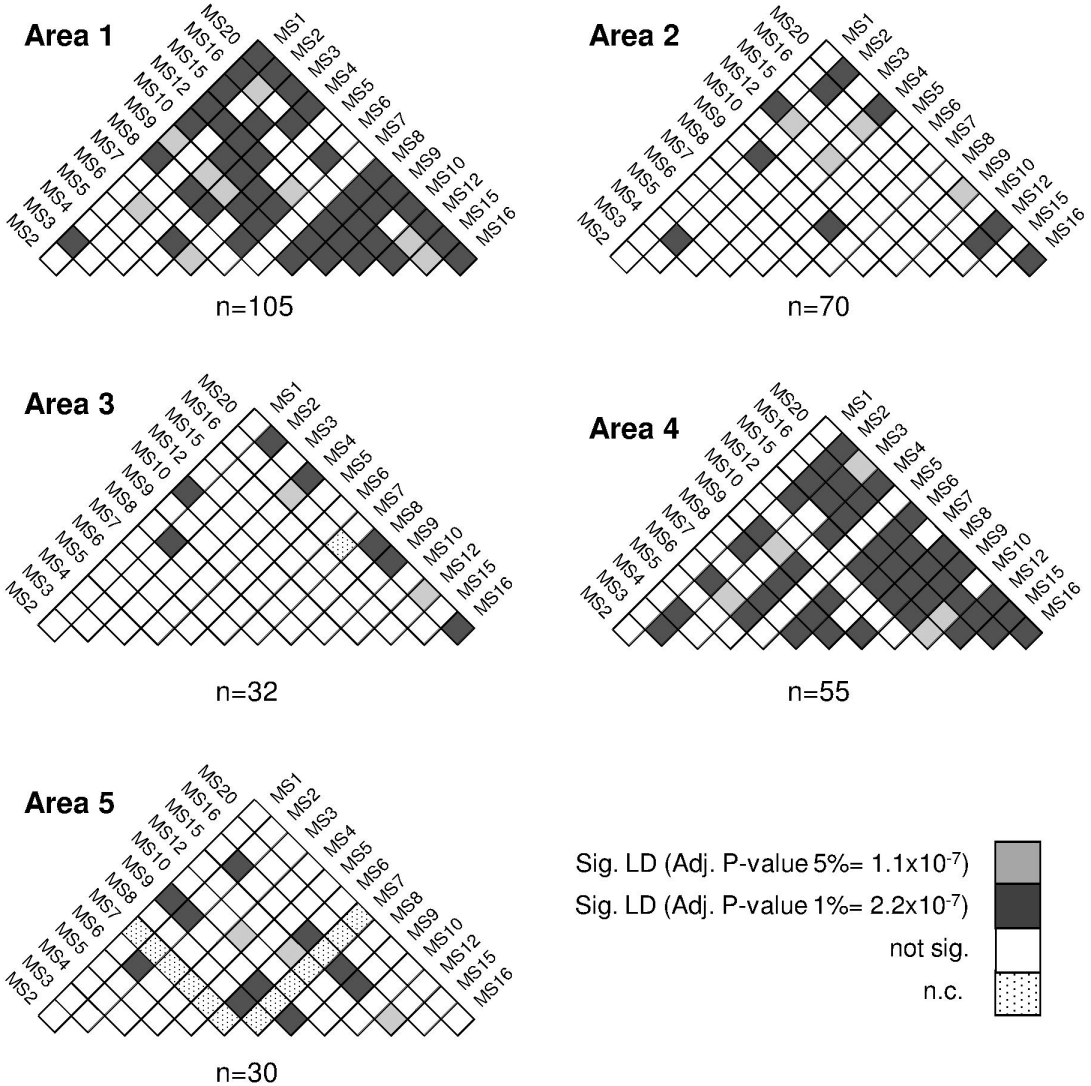
Pairwise-locus linkage disequilibrium analysis by study areas. Significant association between alleles at pairs of loci in each isolate (LD) was tested using FSTAT. *p*-Values were adjusted after Bonferroni corrections and depicted by a colour as described in the legend. Isolates with missing alleles where included in the analysis.

In all study areas except for A5, multilocus linkage disequilibrium (LD) was found when all isolates (MOI≥1) or only monoclonal (MOI = 1) isolates were analysed ($I_{A}^{s}$ = 0.08–0.49, for all areas except A5: *p*<0.0001) (Table 3). LD remained in those areas when only unique haplotypes were considered for the analysis ($I_{A}^{s}$ = 0.08–0.17, *p*<0.0001). The number of isolates decreased drastically when only monoclonal infections were considered (in A3, from 10 polyclonal isolates to 3 monoclonal isolates). Further LD analysis was performed at village-level only for those villages with more than four isolates. LD was found in villages within A1 (MN, SR and LP, $I_{A}^{s}$ = 0.18–0.21 *p*<0.0001); A2 (VS, $I_{A}^{s}$ = 0.38 *p*<2.0x10^-04^); A3 (VA, $I_{A}^{s}$ = 0.39 *p*<1.0x10^-05^) and A4 (SC, $I_{A}^{s}$ = 0.52 *p*<0.0002). In contrast to the multilocus LD analysis, the pairwise LD analysis was performed by including isolates with missing alleles and showed presence of LD in all areas. The pairwise LD was found mainly between loci located in different contigs therefore the LD found among loci within the same contigs did not alter the outcome (Fig 4).

**Table 3:** Multilocus linkage disequilibrium assessed using the Index of association ($I_{A}^{s}$).

Area	All isolates			MOI = 1			Unique haplotypes		
	# isolates	$I_{A}^{s}$	*p*-value	# isolates	$I_{A}^{s}$	*p*-value	# isolates	$I_{A}^{s}$	*p*-value
A1	60	0.15	<0.0001	59	0.19	<0.0001	46	0.10	<0.0001
A2	16	0.08	<0.0001	10	0.05	0.0078	16	0.08	<0.0001
A3	10	0.24	<0.0001	3	0.83	0.0002	9	0.17	<0.0001
A4	38	0.49	<0.0001	36	0.49	<0.0001	12	0.14	<0.0001
A5	4	0.01	0.58	3	0.27	0.11	4	0.01	0.58
All areas	128	0.12	<0.0001	111	0.17	<0.0001	87	0.06	<0.0001

LD analysis for each area at three levels: all isolates, monoclonal isolates only (MOI = 1) and unique haplotypes only. *p*-Values to test the null hypothesis of linkage equilibrium. Isolates with missing alleles were excluded from the analysis.

### Genetic structure

**Fig 5:**
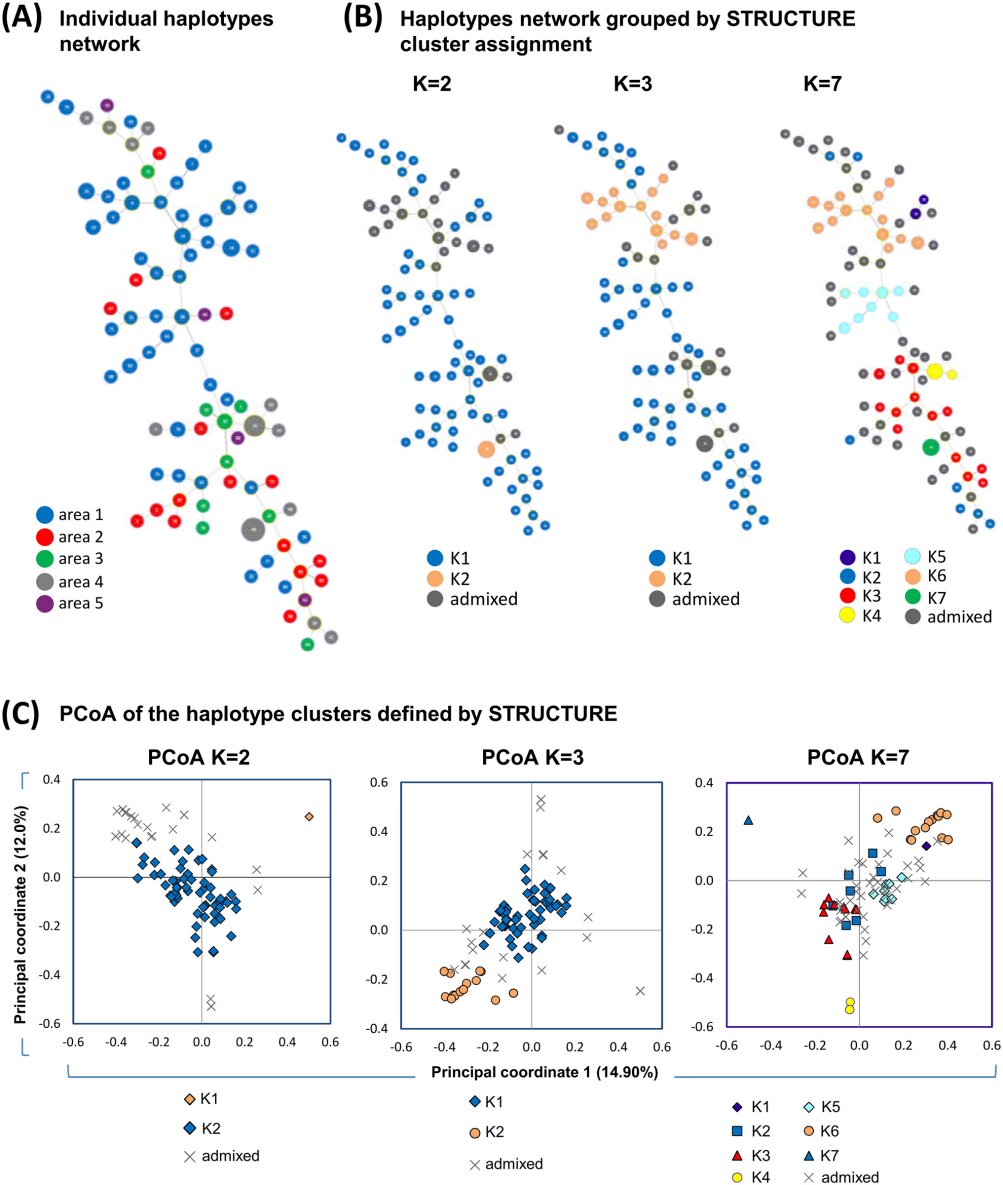
Phylogenetic relationship and PCoA of the *P*. *vivax* haplotypes arranged by the geographic origin and the genetic clustering. (A) Network analysis displaying the phylogenetic relationship of the *P*. *vivax* haplotypes. The haplotypes are coloured following the area colour code displayed in the legend. The size of the nodes denotes the number of haplotypes transformed in logarithmic scale. (B) Phylogenetic relationships and PCoA of the isolates assigned into 2, 3 and 7 genetic clusters following STRUCTURE criteria. The nodes were coloured to discriminate their assignment to a genetic cluster (*K*1, *K*2…) or as an admixed haplotype and the node’s size varied as described previously. When *K* = 3 only 2 clusters are described (*K*1 and *K*2) since no isolate achieved effective assignment to *K*3. (C) The genetic distances among the haplotypes are described by the PCoA graphs and coloured according to the assignment to a specific genetic cluster or as admixed haplotypes. Percentages in the parentheses indicate the proportion of total variation explained by each principal coordinate.

**Fig 6:**
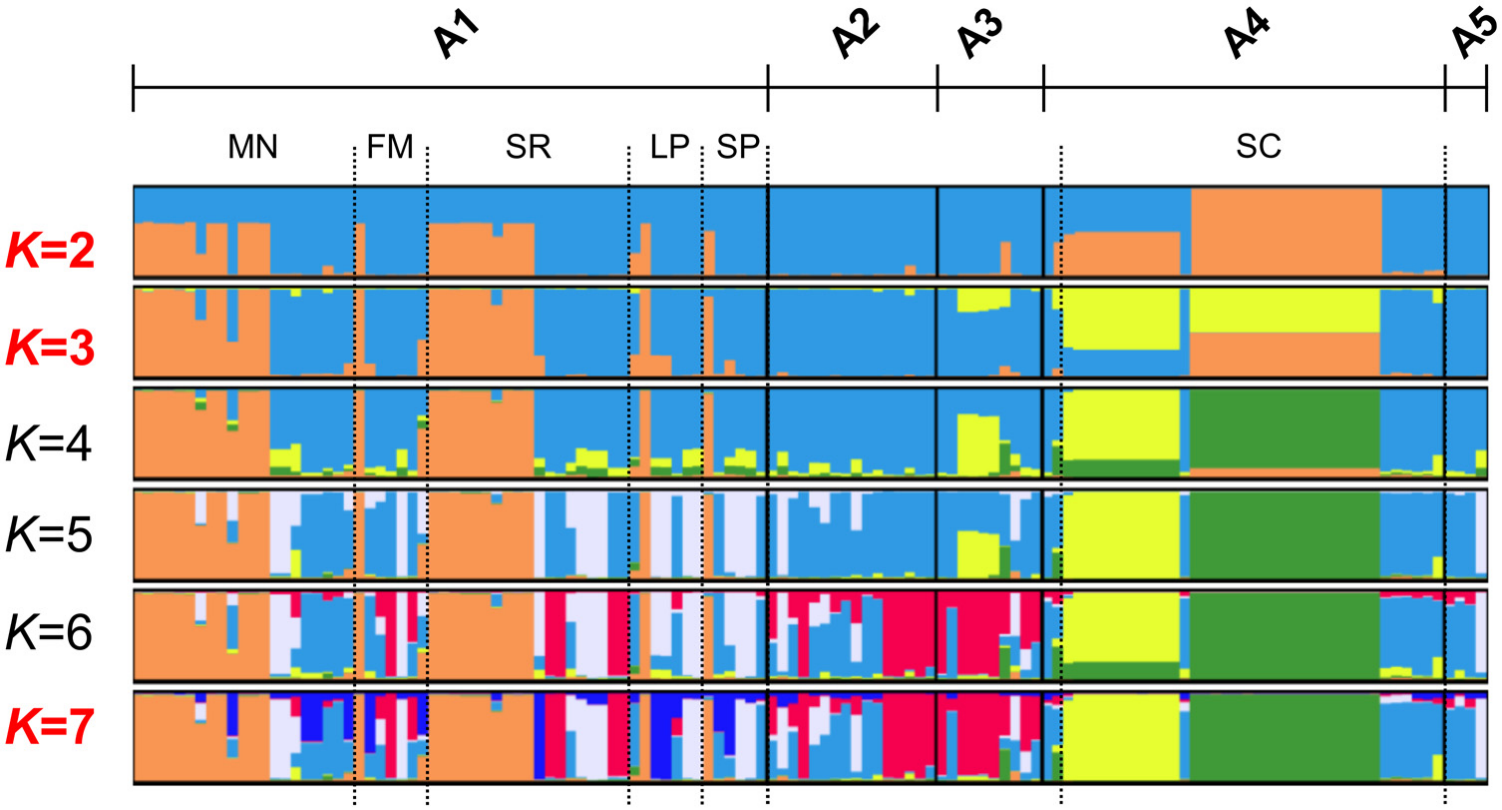
Genetic clustering analysis by STRUCTURE. The graph depicts the clustering models when the isolates were assigned into 2 to 7 clusters (*K* = 2, 3 and 7 being the best models). Each isolate is represented by a single vertical line broken into *K* coloured segments, with lengths proportional to each of the *K* inferred clusters.

87 unique haplotypes were found (52.9% of them in A1) in 128 isolates without allelic missing data (Fig 5A; list of haplotypes in S3 Table). The two most frequent haplotypes were found 18 and 11 times (both in A4), followed by 11 haplotypes found 2–4 times (10 from A1 and 1 from A3) and 74 haplotypes were present only once (Fig 5A). Haplotypes were not shared between areas and only shared within the villages of A1. The haplotypes were grouped into 9 to 13 genetic clusters when the eBURST criteria was set to 8 to 13 loci with identical alleles. When the criteria was set to 7 loci, eBURST assigned the parasite population into 3 genetic clusters (*PHI*_PT_ among clusters = 0.25, *p* = 0.001) and 6 singletons, where one cluster accounted for 91% (116/128) of the isolates. The *K*-means clustering divided the population into 2 and 3 genetic clusters and significant genetic differentiation between clusters was found (*Rho* 0.35 and 0.23, respectively). STRUCTURE analysis was performed to infer cluster assignment including only isolates with up to 3 missing alleles and with known haplotypes. Using STRUCTURE results HARVESTER predicted the most likely number of clusters being *K* = 2, followed by *K* = 3, and *K* = 7 (Fig 6). Using a threshold of 85% for the assignment of group representatives to each cluster, 70–63% of the isolates were assigned to clusters for *K* = 2, *K* = 3 and *K* = 7 and the remaining isolates were assigned as admixed parasites (Fig 5B). The AMOVA analysis revealed high differentiation between these genetic clusters (*PHI*_PT_ = 0.26–0.59) (Table 1) which were graphically displayed using PCoA (Fig 5C). When the number of clusters was set to *K* = 2, parasites from all areas belonged to cluster 2 (K2: *He* = 0.76; $I_{A}^{s}$ = 0.06 *p*<0.0001) while 18 isolates from San Carlos village belonged to cluster 1 (K1: all the isolates shared the same haplotype) (Fig 5B). Hierarchical structure was subsequently found within cluster 2 where two sub-clusters were found (*PHI*_PT_ = 0.20, *p*<0.0001), A1 contained one cluster and hybrid samples while the other cluster was present in all the other areas (S2 Fig). When *K* = 3, the former 18 isolates from San Carlos and 11 additional isolates from San Carlos were classified as admixed parasites where their genetic composition shared part of the cluster 1 or 2 with a third cluster. No parasites with >85% ancestry belonging to the third cluster were found within our study. Cluster 1 and 2 contained parasites from all areas (*He* = 0.77 and 0.35;$\;I_{A}^{s}$ = 0.05 and 0.11, for both *p*<0.0001). The extent of genetic diversity and LD within the clusters decreased when *K* = 7 (*He* varied from no diversity to 0.74, median *He* = 0.35) and linkage disequilibrium was found for all the clusters (*p*<0.01).

### Network and gene flow

The minimum spanning tree in Fig 5A displayed the phylogenetic relationship among the parasites coloured by its geographic origin. Besides most A1’s parasites were related to other A1’s parasite (probably due to its larger sample size), all the rest of A1 parasites shared a phylogenetic relationship with parasites from other areas. Among A2 and A3 haplotypes, most A2 haplotypes diverged from A3 haplotypes but almost none from A2 to A3. By the way, the two largest clusters of haplotypes from A4 have diverged from parasites similar to the ones found in A3 while all A5 parasites have diverged from parasites from other areas.

**Fig 7:**
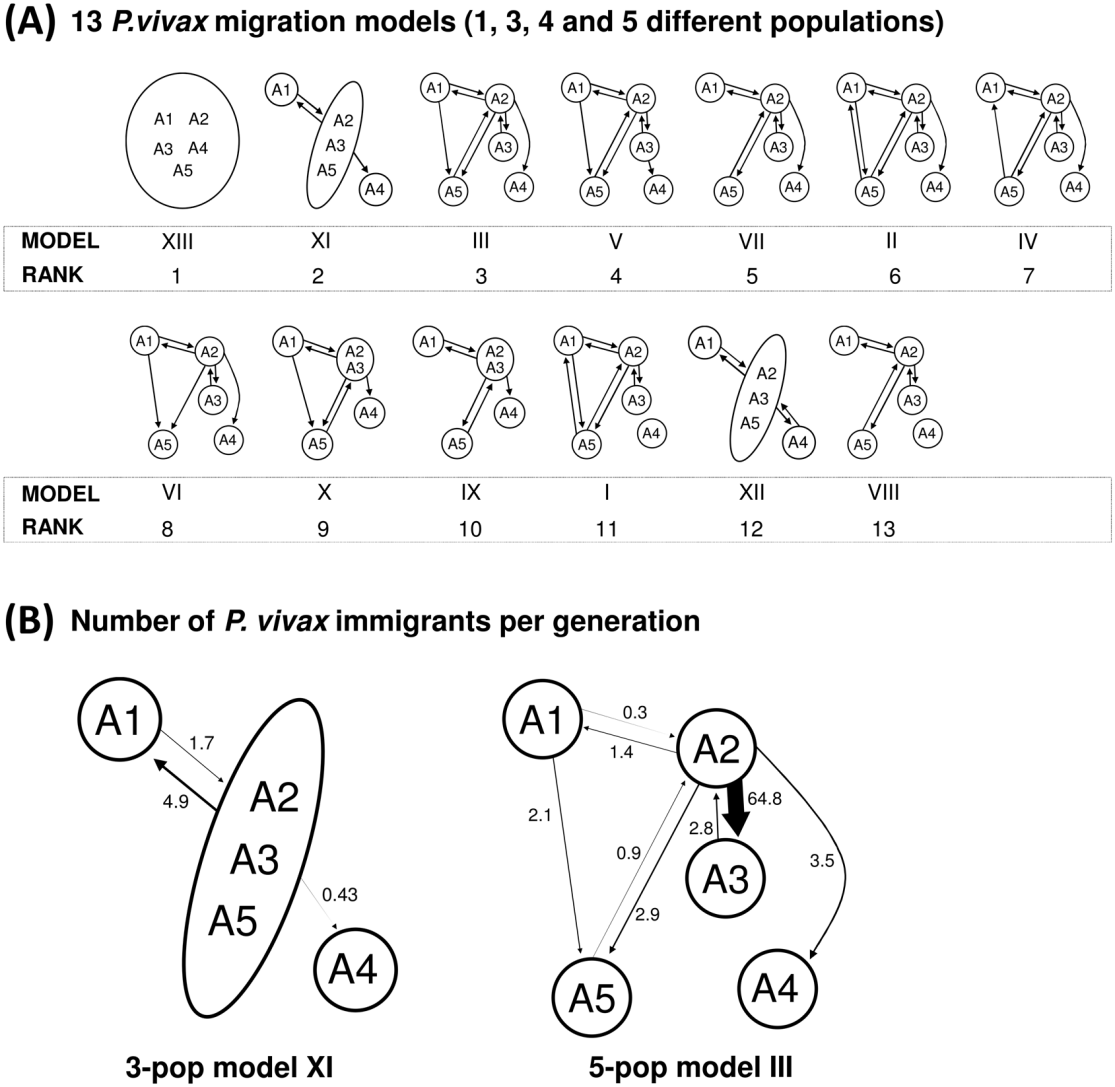
Comparison of 13 parasite migration models and summary estimates of gene flow. **(A)** The study areas were pooled as an unique population and in 3–5 populations. The arrows represent the direction of migration and the models are displayed by its rank order. The marginal log-likelihoods and LBF used to rank the models are tabulated in S4 Table. The model XIII (panmixia) has the highest model probability (>0.99) while all the rest have near zero probability. **(B)** The estimated number of immigrants per generation are described for the models XI and III. The thicknesses of the arrows are proportional to the number of immigrants. The Θ and *M* estimates for the models XIII, XI and III are tabulated in S5 Table.

The gene flow was assessed evaluating parasite migration models which relied on the combined knowledge of the genetic structuring of the parasite population and the known human mobility patterns (Figs 1B and 7A). Thirteen migration models were evaluated through Bayesian analysis (marginal likelihoods and LBF of all the models tabulated in S4 Table). The high gene flow rates among all areas denoted the model XIII as the best model (log mL = -47467.4, prob>0.99), which describes a single panmictic population (random mating among the parasites from all the five areas) with an effective population size of 6,891 haplotypes (credibility interval 95% 4,144–9,640). Considering the genetic substructuring and asymmetric human mobilization observed for most of the areas which contrast with the panmictic model, the 2^nd^ and 3^rd^ best models which consider 3 and 5 populations with asymmetric migration were also explored in detail (Fig 7B). The 3-population model XI suggests A2, A3 and A5 as a panmictic unit with unequal migration among A1 to A2/3/5 and few parasite moving from A2/3/5 to A4 (*Nm*<1). The 5-population model III adds unidirectional gene flow from A1 to A5 which is in line with the phylogenetic results (Figs 5A and 7B). For Model III the highest rate of gene flow was found between the A2 and A3, areas which contain parasites with common genetic characteristics (PCoA and cluster analysis), have the highest multiplicity of infection rates compared to the other areas and is consistent with the current people’s mobilization patterns (Figs 1B, 2A, 3A and 7B). Other models were evaluated were A4 was treated as a fully isolated area or with bidirectional gene flow but these models had the lowest probabilities (Fig 7A and S4 Table). However, model V described unidirectional gene flow from A3 to A4 with a high number of migrants (*Nm* = 5.6) probably due to related parasite among A3 and A4.

### Bottleneck

BOTTLENECK analysis using 10 polymorphic markers showed a significant number of microsatellites had an excess of *He* in areas A1, A2 and A3 under IAM (*p*<0.002) and TPM (only for A2 and A3, *p*<0.04), indicating a recent bottleneck event and a deficiency of *He* for A4 under SMM (*p* = 0.002), possibly indicating a rapid expansion (S3 Fig). However, A1 also presented contrasting *He* deficiency under SMM (*p* = 0.002). A5 was not included in the analysis (n = 4). Similar results were obtained when the analysis was performed separately for perfect and imperfect microsatellites as shown in S3 Fig The presence of excess and deficiency of *He* in A1 was further investigated by grouping the isolates by village (whenever the sample size was sufficiently large): excess of *He* in all villages was found but in addition deficit was also found in Manacamiri village (MA: IAM for imperfect MS: excess *p* = 0.008 and deficit *p* = 0.04; SMM for perfect MS: excess *p* = 0.06 and deficit *p* = 0.03).

## Discussion

The present study provides a very comprehensive dataset on population genetics and gene flow analysis of *P*. *vivax* parasites circulating in the Peruvian Amazon. We determined and compared the genetic diversity and multiplicity of infection in five areas in and around Iquitos. We further unravelled the population structure by assessing the LD, genetic differentiation and determined the most likely number of genetic clusters and the genetic relationships among parasites. Multiple gene flow models were assessed determining the parasite migration patterns that may affect the genetic structuring. Moreover the occurrence of recent bottleneck events as result of recent malaria control programs were also explored. The findings support a sub-structured parasite population with a predominant clonal propagation and revealed that Iquitos city (A2) is the source of parasite spreading for all the other areas due to socio-economic patterns. Recent bottleneck events were found only in areas where intervention control programs were carried before we started the sample collection.

### Multiplicity of infection

MOI, the average number of distinct parasites infecting an individual in a specific area, has been used as a proxy of malaria transmission [12], and it provides information about the configuration of the parasite population [10]. Overall, monoclonal *P*. *vivax* infections were the most frequent, confirming previous studies from the Peruvian Amazon [6, 14, 51]. Areas with some degree of isolation, such as areas A1 and A4, had mainly monoclonal infections, whereas the areas close to the Iquitos-Nauta road (A2 and A3) had polyclonal infections with 2 or 3 different clones and a similar proportion of monoclonal and polyclonal infections. This difference may be explained by a higher mobility rate of the people from A2 and A3 leading to a higher probability of being infected with distinct parasites. In areas with limited gene flow, A1 and A4, frequent mating between genetically identical or very related parasites may increase the odds that a person is re-infected with the same “clone”, which would result in a low MOI [52]. Moreover, the MOI will be affected by the patterns of hypnozoite activation where the probability of having a homologous activation may be higher in areas with one or few circulating clones compared to areas where unrelated clones are circulating.

### Genetic diversity

Previously, varying levels of genetic diversity have been reported in endemic settings of the Peruvian Amazon revealing different transmission patterns [6, 14, 15, 51]. Multivariate analysis (AMOVA) indicated that the major source of the genetic variation was due to variation within villages instead of between areas. The greater genetic variation within the villages may be explained by the coexistence of different haplotypes within the villages as results of gene flow, genetic drift and/or a large hidden *P*. *vivax* reservoir but also by the high mutability of the microsatellites [53] or overestimation of the number of different haplotypes. The degree of polymorphism of the microsatellites or genotyping errors due to technical artefacts (false alleles) may influence the accuracy of defining the presence of one or more haplotypes within an infection [54].

In our study the levels of genetic diversity remained at intermediate levels with no substantial differences between areas. The high rates of gene flow found may have increased the levels of *He*. In the present study we reported coexistence of LD and extensive diversity also reported in other studies [55–57]. In the case of repeat-sequence in tandems like the microsatellites, the level of genetic polymorphism (‘genetic diversity’) may be maintained or increased due to the appearance of new alleles by mutational events during replication in the host cells without affecting the LD [55, 57]. Considering long-term *P*. *vivax* infections in the Peruvian Amazon the rate of allele mutations occurring within the host needs to be further explored. On the other hand, the extent of LD could be overestimated due to some of the recruited vivax patients were living in the same household [57]. The presence of LD and relatively high genetic diversity may be also favoured by a scenario where inbreeding of few sympatric divergent parasites is frequent [58].

### LD and predominant clonal propagation

Similarly to other *P*. *vivax* populations from South America [11, 53, 58–62], the presence of LD and low MOI indicated a clonal propagation type in our study population. The low malaria transmission and/or the long-term vivax infections in the Peruvian Amazon may also favour the predominant clonal propagation [6]. Passive clonality due to restrained diversity and low gene flow, a different scenario to what we found in the present study, may not be the only type of clonal propagation occurring in the study areas. Tibayrenc and Ayala (2014) coined the ‘in-built, active clonality’ whereas *P*. *falciparum* despite the possibility of recombination with sympatric unrelated clones *P*. *falciparum would* prefer self-fertilization in order to gain biological and evolutionary adaptations to its environment [52]. The high rate of gene flow and high variability within the study villages drive us to consider that *P*. *vivax* may have also in-built, active clonal behaviour in order to take advantage during its adaptation to the environment/hosts. Further study is needed to verify the in-built clonal behaviour and its impact on the epidemiology.

### Genetic relatedness

Genetic clustering approaches revealed the presence of at least two or three independent parasite clusters in our study population. We rerun STRUCTURE within one cluster of the cluster when *K* = 2 to: (1) look for hierarchical structure and; (2) to avoid misleading cluster assignment [37]. Sub-structured parasite population was confirmed in areas A1 and A4 and possibly misleading initial STRUCTURE assignment of the clusters (this is also graphically detected in Fig 5C). The cluster 1, which contained 18 isolates carrying the same haplotype, may influence the misleading assignment: STRUCTURE tends to group strongly related samples into one cluster and the rest of samples are assigned in a large cluster [37].

Little to moderate differentiation among the parasites was found in the urban areas A2, A3 and A5 (“urban cluster”) where continuous human mobilization and parasite gene flow on the Iquitos-Nauta Road occur. Conversely, different clusters of parasites were found circulating in the area A1 and A4 increasing the levels of genetic differentiation. In the San Carlos village (A4) was detected a group of parasites circulating only within this village (Fig 6). Major divergence of these parasites may have occurred due to the limited gene flow, genetic drift, bottleneck events, selection and/or recombination with imported parasites not being sampled in this study. The LD and the deficiency of *He* detected in A4 confirmed the rapid expansion of these clones with clustered transmission previously described after 2-year follow up [6].

### *P*. *vivax* transmission in the Peruvian Amazon

Determining the transmission patterns is a priority for the implementation of control and elimination programs [63]. Our initial analysis revealed no genetic isolation of the study areas despite the geographic distance indicating that exist gene flow. Overall, few private alleles were found among areas where A3 and A5 had the lowest numbers supporting gene flow among the areas where A3 and A5 are beneficiated with more immigrants (parasites). However due to sample size bias on the calculation of private alleles we further used a Bayes approach where our sample size was not anymore an issue. We evaluated 13 migration models, using a Bayes approach based on the coalescence theory, which were proposed under the assumptions of current human mobilization and the genetic structuring data. The panmictic model was inferred as the best model, which is in line with the AMOVA results (less genetic differentiation among areas compared to within the areas). However, due to geographic constrains it is unlikely that people mobilizes to every area while parasites mate randomly. Possibly our genetic data was not strong enough to distinguish the best model: i.e. recent bottleneck events may affect the analysis [45]; however still we were able to recover two models that proposed a better explanation regarding the transmission dynamics and genetic structuring. Both models (XI and III) agreed that area A2 (for model XI: A2/3/5) is the source of parasites spreading for all the other areas: A2 comprises Iquitos city, place where people from A1 and A3 transmutes to every day, crossing the Nanay river by boat or going by car/bus/motorbike through the Iquitos-Nauta Road, for economic activities (Figs 1A and 1B and 7B). The influx of people from A1 to A2 is drives most of the parasite influx among these areas where people from A1 may be infected in A2 then importing parasites from A1 to A2 when they return to their households. Model XI described a panmixia between A2, A3 and A while the model III showed that most of migration occurs between A2 and A3, favoured by the proximity and the road (no geographic barrier) in line with the shared genetic parasite characteristics found previously. In line to the phylogenetic analysis, the model III described that A5 have influx of parasites from A2 which could occur during the visit of people from A5 to A1 on weekends to sell their products in the markets of Iquitos. Model III also described influx of parasites from A1 to A5 despite the Nanay river isolate both areas. High rates of parasite migration from A2 to A3 and A5 is in line with low (or none) number of private alleles recorded for A3 and A5. Regarding to area A4, is it known that occasionally people from A4 (especially from San Carlos) travels through the Itaya river to the A2 to sell their products and that may explain the importation of parasites from A2 to A4. As mentioned before, some parasites from A4 are related to parasites from A3, which in turn are highly related to A2 parasites. The relatedness of A4 and A3 parasites (Network analysis) and migration of parasites from A3 to A4 (referred only in model IV) may have occur by unknown human migration patterns or events where the vector mobilizes among these areas or genetic divergence of A3 parasites into the current A4 parasites. We have documented parasite transmission from A2 to the other areas but not significant immigration of parasites towards A2 which explains why A2 has the smallest population size (number of haplotypes) compared to the other areas (S5 Table) and why we still found significant pairwise genetic differentiation against A1 and A4. In addition to the low rate of immigrants to A2, recent bottleneck events may have also negatively affected the effective population size in A2.

### Bottleneck events after intervention program

By describing the parasite structure, genetic diversity and dynamics, population genetics can also contribute in assessing the impact of an intervention [10]. Before our sample collection in 2008, the PAMAFRO project which involved campaigns of malaria prevention and control program with active case detection and treatment as well as distribution of insecticide-treated mosquito nets was carried out in Loreto (including all our study areas except for area A4), resulting in a 49% drop of the incidence of clinical vivax malaria from 2005 to 2008 [20]. The expected impact of the intervention on the parasite population besides lowering the malaria incidence would be a reduction of the effective parasite population size, the so called bottleneck effect. Since no data prior to the intervention on the effective parasite population size were available, we performed a retrospective analysis looking for recent bottleneck effect. A parasite population having experienced a recent bottleneck shows a faster decline in the number of alleles compared to a *He* reduction because rare alleles will be lost with little influence on the *He* [47]. The predominant clonal propagation found in this study did not affect the Bottleneck analysis since moderate *He* and significant intra-area genetic variation were found. Only areas with *n*>15 isolates (S3 Fig) were used for the analysis to increase the resolution power as described by Luikart *et al*. [64]. In this study, bottleneck events were detected in all areas where control interventions were implemented. San Carlos village did not benefit of any control activity before the sample collection, and no bottleneck but rather a rapid clonal expansion was observed. This is the first report of bottleneck events for *P*. *vivax* population in the Peruvian Amazon following the implementation of prevention and control activities. Noteworthy that the reduction of malaria cases in Peru lasted only until 2011 coinciding with the finalization of the PAMAFRO project and since then there has been an steady increase of malaria cases: i.e. Loreto region reported 11,779 vivax malaria cases in 2011 and 60,566 in 2014 [3, 65]. Further and continuous monitoring of the population structure and dynamics of the parasite population is necessary to understand the factors that are involved in the evolution of malaria in the Peruvian Amazon. The detection of recent bottlenecks in the parasite population could be used as complementary tool to measure the efficacy and impact of malaria control programs.

In conclusion, we have elucidated the population genetics of *Plasmodium vivax* in a large geographical area in and around Iquitos, the main socio-economic capital city of the Peruvian Amazon. We have shown the use of a Bayes approach to infer the gene flow pattern among our study areas and the detection of the reduction of the population size as a result of a control program. The knowledge about the routes of malaria transmission (gene flow) and the effect of control policies on the parasite population is a priority from the public health perspective as well for the formulation of future control policies and assessment of current control/elimination strategies.

## Supporting Information

S1 Table Distribution of *Plasmodium vivax* isolates by study areas and villages . The number of isolates is described for each village.
(PDF)

S2 Table Allelic size range, expected heterozygosity (*He*) and efficiency of MS-PCR amplification of each locus. The allelic size range is described in base pairs (bp). In this table, the level of polymorphism of each locus was described as the expected heterozygosity (*He*) calculated for each marker using pooled isolates from all areas. The efficiency of amplification was calculated as the percentage of isolates with positive PCR amplification for a specific locus over the total number of analysed isolates (n = 292).
(PDF)

S3 Table List of the 87 unique *P*. *vivax* haplotypes found in the present study. (PDF)

S4 Table Marginal log-likelihood (log mL) and log Bayes factors (LBF) values used to rank the models in Fig 7. The models were constructed considering 5, 3 and 1 populations evaluating from 1 to 14 combined parameters (Θ and M) using MIGRATE-N.
(PDF)

S5 Table Estimates of the migration parameters for the three best migration models: mutation scaled population size (Θ) and mutation scaled migration rates (*M*). (PDF)

S1 Fig Mantel test for Isolation-by-Distance. Mantel test for Matrix Correspondence was performed using GenAlEx (1) to test the occurrence of a positive correlation (*Rxy*>0) between the genetic *PHI*_PT_ matrix and geographic distances, so called the Isolation-by-distance (IBD) hypothesis. The genetic and geographic distance were not correlated (*Rxy* = -0.41 *p* = 0.17).
(PDF)

S2 Fig Detection of hierarchical structure in one of the clusters detected in the initial STRUCTURE analysis. (PDF)

S3 Fig Detection of recent bottleneck or expansion events in the parasite population. (PDF)
