# Supplementary material for: Population Genetics of Plasmodium vivax in the Peruvian Amazon
Source: PLoS Negl Trop Dis. 2016 Jan 14;10(1):e0004376. doi: 10.1371/journal.pntd.0004376 (PMC4713096; doi:10.1371/journal.pntd.0004376)
Supplement: S1 Table — . The number of isolates is described for each village. (PDF) [file pntd.0004376.s001.pdf]

S1 Table. Distribution and characteristics of *Plasmodium vivax* isolates by study areas and villages. The composition of each study area is described in this table. The number of isolates (total n. of isolates=292), type of location, malaria control and/or intervention activities carried before our study sampling, median parasitemia (trophozoites/µl), median age and male/females ratio are described for each village and/or area.

Area	Village	Abbrev.	Code	Isolates (n)	Loca-tion	Inter-vention	Parasitemia	Age (in years)	Males:Females
A1	Manacamiri	MN	1	46	rural	yes			
	Fray Martín	FM	2	8	rural	yes			
	Santa Rita	SR	3	28	rural	yes			
	Lupuna	LP	4	15	rural	yes			
	San Pedro	SP	5	8	rural	yes			
	**total A1**			105 (36%)			3280 (IQR 1173.5-5881.5)	15 (IQR 10-37)	1.14
A2	Porvenir	POR	6	4	peri-urban	yes			
	Aguaje	AG	7	2	peri-urban	yes			
	Pampachica	PP	8	1	peri-urban	yes			
	Bella Luz	BE	9	2	urban	yes			
	Claveles	CL	10	1	peri-urban	yes			
	Cardozo	CAR	11	1	urban	yes			
	Palmeras	PAL	12	1	urban	yes			
	Las Rosas	LR	13	2	peri-urban	yes			
	Villa Disnarda	VD	14	2	urban	yes			
	Juan Carlos del Aguila	JCA	15	2	peri-urban	yes			
	Mercedes	ME	16	2	peri-urban	yes			
	Amistad	AMI	17	3	peri-urban	yes			
	Progreso	PRO	18	1	peri-urban	yes			
	San Pablo de la Luz	SPL	19	2	peri-urban	yes			
	America	AME	20	1	urban	yes			
	Simon Bolivar	SB	21	3	peri-urban	yes			
	Camelias	CAM	22	1	peri-urban	yes			
	Terminal	TE	23	4	urban	yes			
	Villa Selva	VS	24	27	urban	no			
	Ficus	FI	25	2	urban	yes			
	San Julian	SJ	26	5	urban	yes			
	Quistococha	QUI	27	1	rural	yes			
	**total A2**			70 (24%)			4740 (IQR 2039-5570)	25.5 (IQR 13-42)	1.06
A3	Cruz del Sur	CS	28	1	rural	yes			
	Delfines	DE	29	4	rural	yes			
	Unión Progreso	UP	30	2	rural	yes			
	Peña Negra	PN	31	1	rural	yes			
	Varillal	VA	32	21	rural	yes			
	5 de Abril	AB	33	3	rural	yes			
	**total A3**			32 (11%)			2494.5 (IQR 870-5042)	17 (IQR 12-28)	1.13

Area	Village	Abbrev.	Code	Isolates (n)	Loca-tion	Inter-vention	Parasitemia	Age (in years)	Males:Females
**A4**	El Milagro	MI	34	1	rural	yes			
	Villa Buen Pastor	VBP	35	15	rural	yes			
	San Carlos	SC	36	36	rural	no			
	3 de Octubre	OC	37	3	rural	yes			
	**total A4**			55 (18.8%)			3458 (IQR 1258.5-5280)	21 (IQR 8-35)	1.12
**A5**	El Dorado	DO	38	3	rural	yes			
	13 de Febrero	FE	39	5	rural	yes			
	Paujil zone I	PAI	40	2	rural	yes			
	Paujil zone II	PAII	41	2	rural	yes			
	Ex Petroleros	PE	42	5	rural	yes			
	Nuevo Horizonte	NH	43	3	rural	yes			
	San Lucas	SL	44	4	rural	yes			
	El Triunfo	TRI	45	4	rural	yes			
	La Habana	LH	46	1	rural	yes			
	Cahuide	CAH	47	1	rural	yes			
	**total A5**			30 (10.3%)			3413.5 (IQR 1454-5502)	21.5 (IQR 12-39)	1.73
