# Supplementary material for: Population Genetics of Plasmodium vivax in the Peruvian Amazon
Source: PLoS Negl Trop Dis. 2016 Jan 14;10(1):e0004376. doi: 10.1371/journal.pntd.0004376 (PMC4713096; doi:10.1371/journal.pntd.0004376)
Supplement: S2 Table — The allelic size range is described in base pairs (bp). In this table, the level of polymorphism of each locus was described as the expected heterozygosity (He) calculated for each marker using pooled isolates from all areas. The efficiency of amplification was calculated as the percentage of isolates with positive PCR amplification for a specific locus over the total number of analysed isolates (n = 292). (PDF) [file pntd.0004376.s002.pdf]

S2 Table. Allelic size range, expected heterozygosity (*He*) and efficiency of MS-PCR amplification of each locus. The allelic size range is described in base pairs (bp). In this table, the level of polymorphism of each locus was described as the expected heterozygosity (*He*) calculated for each marker using pooled samples from all areas. The efficiency of amplification was calculated as the percentage of samples with positive PCR amplification for a specific locus over the total number of analysed samples (n=292).

locus	range (bp)	*He*	efficiency of amplification
**MS1**	230-248	0.63	95.5%
**MS2**	189-237	0.79	93.2%
**MS3**	181-196	0.63	92.5%
**MS4**	188-215	0.70	89.7%
**MS5**	166-229	0.22	92.1%
**MS6**	209-248	0.86	89.7%
**MS7**	143-167	0.20	79.5%
**MS8**	205-340	0.92	84.9%
**MS9**	144-177	0.82	91.8%
**MS10**	178-268	0.66	84.6%
**MS12**	200-332	0.85	81.8%
**MS15**	232-289	0.85	82.9%
**MS16**	158-374	0.90	71.6%
**MS20**	191-254	0.88	84.9%
