# Supplementary material for: Population Genetics of Plasmodium vivax in the Peruvian Amazon
Source: PLoS Negl Trop Dis. 2016 Jan 14;10(1):e0004376. doi: 10.1371/journal.pntd.0004376 (PMC4713096; doi:10.1371/journal.pntd.0004376)
Supplement: S3 Table — (PDF) [file pntd.0004376.s003.pdf]

S3 Table. List of the 87 unique *P. vivax* haplotypes found in the present study.

Haplotype	MS1	MS2	MS3	MS4	MS5	MS6	MS7	MS8	MS9	MS10	MS12	MS15	MS16	MS20
**1**	1	1	2	6	3	8	1	10	2	4	5	6	11	5
**2**	1	2	2	6	3	6	1	9	7	7	8	5	21	3
**3**	1	2	4	2	2	9	1	6	2	1	10	4	10	13
**4**	1	4	1	3	3	3	1	7	6	6	1	5	13	8
**5**	1	9	2	2	3	6	1	11	7	7	12	8	13	8
**6**	2	1	1	2	3	4	1	7	6	11	1	8	3	6
**7**	2	1	1	2	3	4	1	7	6	11	4	5	8	6
**8**	2	1	1	2	3	4	1	19	6	11	4	8	3	6
**9**	2	1	1	2	3	4	1	21	6	6	4	8	3	6
**10**	2	1	2	2	3	4	1	19	6	6	4	8	3	9
**11**	2	1	3	2	3	3	1	17	6	2	5	5	3	6
**12**	2	1	3	2	3	4	1	7	6	11	4	5	8	6
**13**	2	1	3	2	3	4	1	17	6	11	5	9	3	9
**14**	2	1	3	2	3	4	1	19	6	11	4	8	3	6
**15**	2	1	3	2	3	4	1	19	6	11	4	8	3	9
**16**	2	1	3	2	3	4	1	19	10	11	4	8	8	9
**17**	2	1	3	2	3	6	1	17	10	2	5	5	3	6
**18**	2	4	2	1	1	4	1	8	3	6	1	3	3	4
**19**	2	4	2	2	3	1	1	7	4	11	4	8	16	1
**20**	2	4	2	2	3	1	1	19	6	11	4	8	3	9
**21**	2	4	3	5	3	1	1	7	4	11	9	5	16	9
**22**	2	6	1	1	3	6	1	8	8	6	7	5	19	5
**23**	2	6	1	2	3	4	1	7	4	11	1	8	16	1
**24**	2	6	1	2	3	4	1	19	6	11	1	8	3	9
**25**	2	6	1	2	3	4	1	19	10	11	1	8	8	9
**26**	2	6	1	4	1	3	1	4	10	6	12	5	6	4
**27**	2	6	3	2	3	3	1	5	1	8	10	1	9	9
**28**	2	6	3	4	1	3	1	20	1	8	9	8	3	4
**29**	2	6	3	4	1	3	1	20	1	8	10	8	3	4
**30**	3	4	2	1	3	6	1	8	7	8	5	6	3	6
**31**	4	1	2	1	2	5	1	6	6	7	2	6	3	5
**32**	4	1	2	2	3	7	1	10	7	7	13	6	7	5
**33**	4	1	2	2	3	7	1	10	7	7	14	6	7	5
**34**	4	1	2	2	3	8	2	9	7	7	12	5	8	5
**35**	4	1	2	2	3	8	2	12	7	7	12	5	8	5
**36**	4	1	3	1	2	4	1	8	5	7	4	3	20	6
**37**	4	1	4	2	3	4	1	20	5	7	5	9	3	10
**38**	4	1	4	2	3	4	1	20	6	10	5	9	3	10
**39**	4	1	4	2	3	5	1	22	6	5	13	2	10	7
**40**	4	1	4	2	3	5	1	22	6	10	5	10	3	10
**41**	4	1	4	2	3	7	1	18	5	3	6	6	3	7

**42**	4	1	4	4	3	1	1	6	2	10	11	1	3	3
**43**	4	3	2	1	3	7	1	10	10	7	5	8	4	7
**44**	4	4	2	1	3	7	1	10	5	7	6	8	4	7
**45**	4	4	2	1	3	8	1	11	10	7	6	9	4	7
**46**	4	4	2	2	3	7	1	10	7	7	13	6	7	5
**47**	4	4	2	3	3	8	1	8	6	7	5	8	10	12
**48**	4	4	2	3	3	8	2	8	6	7	5	8	23	12
**49**	4	4	2	3	3	9	1	7	6	7	4	7	22	12
**50**	4	4	2	6	3	7	1	1	10	7	6	6	10	6
**51**	4	4	2	6	3	7	1	2	10	7	6	6	10	6
**52**	4	4	3	6	3	4	1	9	2	7	2	6	19	5
**53**	4	5	3	1	2	1	1	9	5	7	5	10	23	5
**54**	4	5	3	1	2	2	1	7	5	7	4	8	20	5
**55**	4	5	3	2	3	1	1	8	5	10	5	9	16	2
**56**	4	5	3	2	3	2	1	7	5	7	4	8	17	2
**57**	4	5	3	2	3	2	1	7	5	13	4	8	17	2
**58**	4	6	2	1	3	3	2	14	3	5	5	2	15	11
**59**	4	6	2	1	3	7	1	7	9	7	15	5	17	6
**60**	4	6	2	1	3	7	1	11	2	7	3	11	1	6
**61**	4	6	2	1	3	7	1	12	4	11	7	1	2	12
**62**	4	6	2	1	3	8	1	14	9	7	7	6	18	6
**63**	4	6	2	2	3	4	1	8	6	10	2	6	8	7
**64**	4	6	2	2	3	4	1	20	3	10	2	9	8	10
**65**	4	6	2	2	3	4	1	20	4	10	2	9	8	10
**66**	4	6	2	2	3	4	2	10	7	7	5	6	3	3
**67**	4	6	2	4	2	3	1	16	6	10	2	8	3	9
**68**	4	6	2	5	3	3	1	11	7	7	2	2	7	2
**69**	4	6	3	6	3	6	1	11	6	5	4	6	14	10
**70**	4	6	4	4	2	3	1	6	2	9	11	1	3	10
**71**	4	6	4	4	2	3	1	20	2	9	11	9	3	5
**72**	4	8	3	2	3	4	1	8	5	11	2	8	18	10
**73**	4	8	3	2	3	5	1	9	5	10	2	4	18	6
**74**	5	1	2	1	1	3	1	8	4	6	4	3	19	6
**75**	5	1	2	1	3	6	1	9	6	7	5	8	7	7
**76**	5	2	2	2	2	7	1	6	2	7	2	6	10	12
**77**	5	4	1	1	3	6	1	12	6	6	3	7	10	5
**78**	5	4	2	4	2	7	1	13	10	10	13	9	18	7
**79**	5	5	2	3	3	3	1	9	7	7	2	6	5	9
**80**	5	7	3	7	3	1	1	3	7	11	4	6	12	7
**81**	6	1	3	2	3	3	1	15	10	7	5	9	19	7
**82**	6	2	1	1	3	8	1	13	7	7	4	6	10	6
**83**	6	2	2	1	3	8	2	12	7	12	3	5	3	8
**84**	6	2	3	1	3	8	1	6	7	7	4	6	10	6
**85**	6	2	3	1	3	8	1	13	7	7	4	6	10	6
**86**	6	4	2	1	3	8	1	12	7	7	4	8	10	6
**87**	6	5	2	3	3	7	1	13	7	7	2	8	13	7
