# Supplementary material for: Population Genetics of Plasmodium vivax in the Peruvian Amazon
Source: PLoS Negl Trop Dis. 2016 Jan 14;10(1):e0004376. doi: 10.1371/journal.pntd.0004376 (PMC4713096; doi:10.1371/journal.pntd.0004376)
Supplement: S4 Table — The models were constructed considering 5, 3 and 1 populations evaluating from 1 to 14 combined parameters (Θ and M) using MIGRATE-N. (PDF) [file pntd.0004376.s004.pdf]

S4 Table. Marginal log-likelihood (log mL) and log Bayes factors (LBF) values used to rank the models in Fig.7. The models were constructed considering 5, 3 and 1 populations evaluating from 1 to 14 combined parameters (Θ and M) using MIGRATE-N.

Model	n. of populations	n. of parameters	log mL	LBF	Rank
**XIII**	**1 (panmixia)**	**1**	**-47467.4**	**0**	**1**
**XI**	**3**	**6**	**-331455**	**-283988**	**2**
**III**	**5**	**13**	**-721627**	**-674159**	**3**
V	5	13	-756949	-709481	4
VII	5	12	-787236	-739768	5
II	5	14	-791710	-744242	6
IV	5	13	-819337	-771870	7
VI	5	12	-873472	-826004	8
X	4	10	-877141	-829674	9
IX	4	9	-969912	-922445	10
I	5	13	-984735	-937267	11
XII	3	7	-1180472	-1133005	12
VIII	5	11	-1858279	-1810811	13

n. of populations: 1 population: all areas considered as one population (panmixia); 3 populations: A1, A2+A3+A5, A4; 5 populations: A1, A2,A3, A4, A5. Model XIII reached the highest model probability (>99.99%) while the others models close to 0.
