# Supplementary material for: Population Genetics of Plasmodium vivax in the Peruvian Amazon
Source: PLoS Negl Trop Dis. 2016 Jan 14;10(1):e0004376. doi: 10.1371/journal.pntd.0004376 (PMC4713096; doi:10.1371/journal.pntd.0004376)
Supplement: S5 Table — (PDF) [file pntd.0004376.s005.pdf]

S5 Table. Estimates of the migration parameters for the three best migration models: mutation scaled population size (Θ) and mutation scaled migration rates (*M*).

Model	Parameter	Mode	Median	95% credibility interval
XIII (panmixia)	$\Theta_{(A1+A2+A3+A4+A5)}$	5.1	5.23	3.07 - 7.13
XI (3 populations)	Θ_{A1}	1.7	1.83	0.07 - 3.4
	$\Theta_{(A2+A3+A5)}$	0.57	1.03	0 - 2.2
	Θ_{A4}	1.17	1.97	0 - 7.87
	$M_{(A2+A3+A5)\rightarrow A1}$	2.9	2.97	1.07 - 4.67
	$M_{A1\rightarrow (A2+A3+A5)}$	2.97	3.1	0.33 - 5.6
	$M_{(A2+A3+A5)\rightarrow A4}$	0.77	1.57	0 - 2.93
III (5 populations)	Θ_{A1}	0.90	1.30	0 - 2.6
	Θ_{A2}	0.03	0.63	0 - 1.73
	Θ_{A3}	6.57	6.70	4.13 - 8.8
	Θ_{A4}	0.03	0.77	0 - 1.93
	Θ_{A5}	1.43	1.63	0 - 3.07
	$M_{A2\rightarrow A1}$	1.57	1.70	0 - 3.2
	$M_{A1\rightarrow A2}$	0.77	1.37	0 - 5.47
	$M_{A3\rightarrow A2}$	2.50	3.23	0 - 13.27
	$M_{A5\rightarrow A2}$	3.10	3.23	0.87 - 5.33
	$M_{A2\rightarrow A3}$	9.30	9.57	6.93 - 13.33
	$M_{A2\rightarrow A4}$	7.43	7.90	4.93 - 12.4
	$M_{A1\rightarrow A5}$	0.90	1.30	0 - 3.27
	$M_{A2\rightarrow A5}$	1.17	1.57	0 - 3.27
