# Supplementary material for: Population Genetics of Plasmodium vivax in the Peruvian Amazon
Source: PLoS Negl Trop Dis. 2016 Jan 14;10(1):e0004376. doi: 10.1371/journal.pntd.0004376 (PMC4713096; doi:10.1371/journal.pntd.0004376)

S1 Fig. Mantel test for Isolation-by-Distance. Mantel test for Matrix Correspondence was performed using GenAlEx to test the occurrence of a positive correlation (*Rxy*>0) between the genetic PHI_{PT} matrix and geographic distances, so called the Isolation-by-distance (IBD) hypothesis. The genetic and geographic distance were not correlated (*Rxy*=-0.41 *p*=0.17).

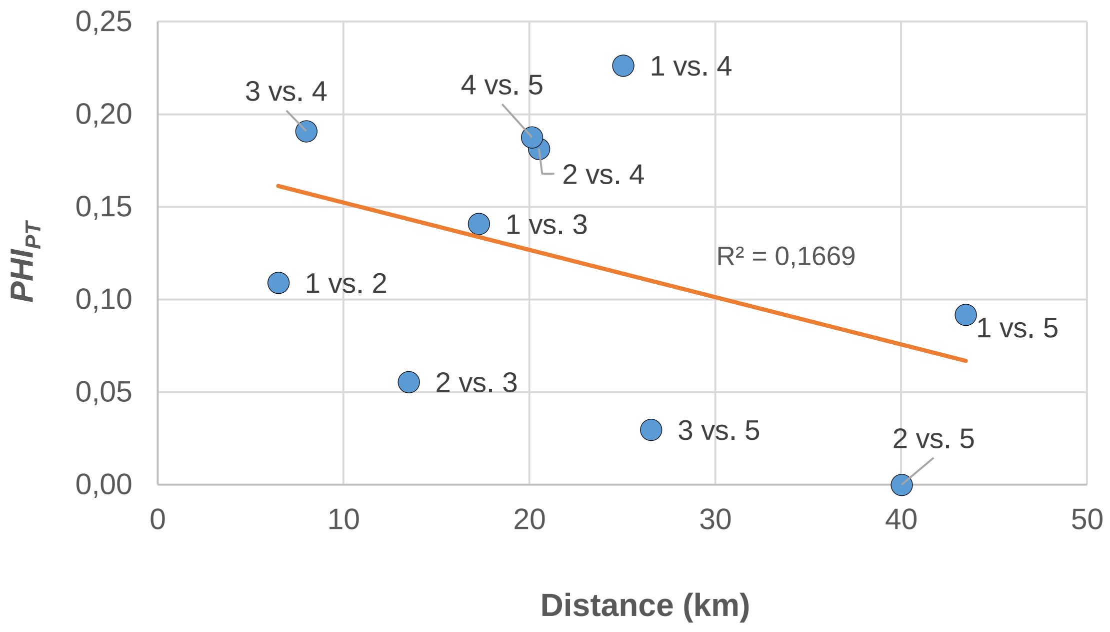

Supplement: S1 Fig — Mantel test for Matrix Correspondence was performed using GenAlEx (1) to test the occurrence of a positive correlation (Rxy>0) between the genetic PHIPT matrix and geographic distances, so called the Isolation-by-distance (IBD) hypothesis. The genetic and geographic distance were not correlated (Rxy = -0.41 p = 0.17). (PDF) [file pntd.0004376.s006.pdf]
