# Supplementary material for: Population Genetics of Plasmodium vivax in the Peruvian Amazon
Source: PLoS Negl Trop Dis. 2016 Jan 14;10(1):e0004376. doi: 10.1371/journal.pntd.0004376 (PMC4713096; doi:10.1371/journal.pntd.0004376)
Supplement: S2 Fig — (PDF) [file pntd.0004376.s007.pdf]

S2 Fig. Detection of hierarchical structure in one of the clusters detected in the initial STRUCTURE analysis.

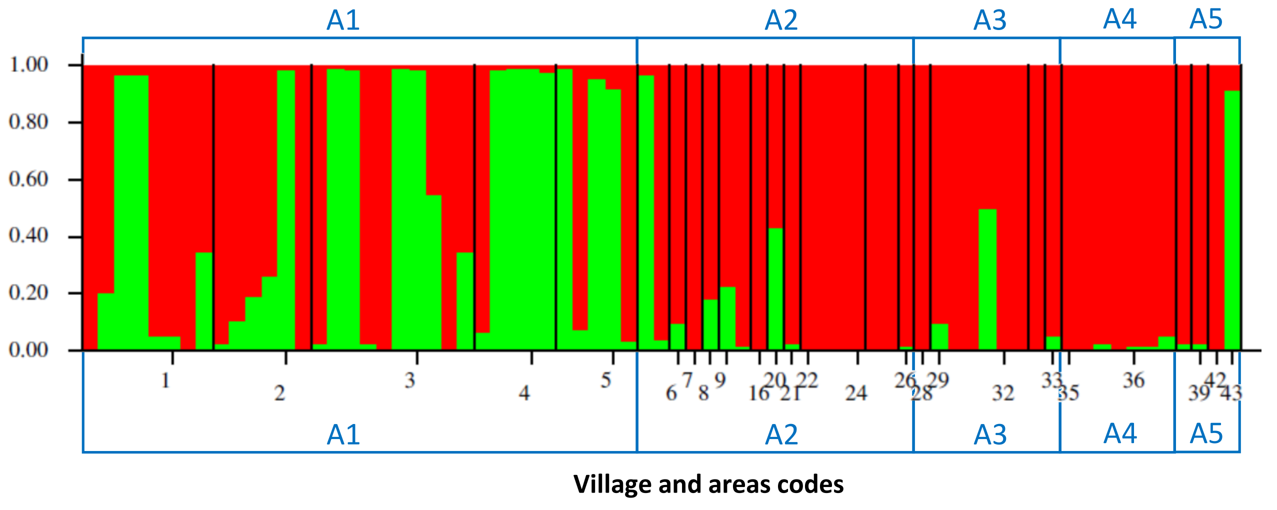

Initial cluster analysis assigned 128 isolates into two clusters and admixed parasites. One of the clusters contained the same haplotype (n=18) and belonged to San Carlos village (A4). The other cluster contained 71 isolates belonging to different areas. This cluster (n=71) was further analyzed for the detection of hierarchical structure and/or incorrect cluster assignment (1). The most likely number of subpopulations was *K*=2. The graph depicts each isolate represented by a single vertical line broken into *K* coloured segments, with lengths proportional to each of the *K* inferred clusters (*Y* axis). The isolates are ordered by its geographical location (number in the *X* axis denotes the village code and area). Areas (A1, A2...) are defined in Fig. 1 and villages abbreviations are detailed in S1 Table.

(1) Kalinowski ST: The computer program STRUCTURE does not reliably identify the main genetic clusters within species: simulations and implications for human population structure. Heredity (Edinb) 2011, 106:625-632.
