# Supplementary material for: Population Genetics of Plasmodium vivax in the Peruvian Amazon
Source: PLoS Negl Trop Dis. 2016 Jan 14;10(1):e0004376. doi: 10.1371/journal.pntd.0004376 (PMC4713096; doi:10.1371/journal.pntd.0004376)
Supplement: S3 Fig — (PDF) [file pntd.0004376.s008.pdf]

S3 Fig. Detection of recent bottleneck or expansion events in the parasite population. The distribution of the number of alleles and *He* has been obtained simulating the coalescence of the microsatellite sequences under three mutational models: IAM, TPM and SMM. The Wilcoxon sign-rank test was used to determine whether a population exhibits a significant number of loci with *He* excess or deficiency (Refs. 1,2). The black cells denote that the parasite population has suffered a recent bottleneck and/or expansion (*He* excess and/or deficit; $p<0.05$) while the white cells denote non-significant evidence. The models were run in BOTTLENECK using 10,000 replications using all 10 microsatellites and later in a partitioned analysis (imperfect and perfect MS analysed separately). The two-Phase model was evaluated using all 10 MS by fractionating the proportion of SMM in TPM by 5, 15, 50, 70 and 95% and a variance of 30. A5 was not included due to small sample size (A5: n=4 haplotypes). Partitioned analysis was run considering 70%SSM in TPM with variance=30 and for 10,000 replicates. Overall results are presented on the smallest table.

BOTTLENECK simulations run under different parameters

Area	Mutational model	*He*	for TPM (%SSM) 5	15	50	70	95	Partitioned analysis Imp.	Perf.
A1 (n=120)	IAM	*def.*							
		exc.	■	■	■	■	■	■	■
	TPM	*def.*							
		exc.							
	SMM	*def.*	■	■	■	■	■	■	■
		exc.							
A2 (n=32)	IAM	*def.*							
		exc.	■	■	■	■	■	■	■
	TPM	*def.*							
		exc.	■	■	■	■		■	■
	SMM	*def.*							
		exc.							
A3 (n=20)	IAM	*def.*							
		exc.	■	■	■	■	■	■	■
	TPM	*def.*							
		exc.	■	■	■	■			■
	SMM	*def.*							
		exc.							
A4 (n=76)	IAM	*def.*							
		exc.							
	TPM	*def.*					■		
		exc.							
	SMM	*def.*	■	■	■	■	■	■	■
		exc.							

(■ = black cell; empty = white cell)

IAM: Infinite allele model; TPM: Two-phase model; SMM: Stepwise mutational model. *exc.*= Significant *He* excess; *def.*= Significant *He* deficiency

Partitioned BOTTLENECK analysis: *Imp.*= MS with imperfect and/or interrupted repeats. *Perf.*= Ms with perfect repeats

Overall BOTTLENECK simulation results

	A1	A2	A3	A4
IAM	*exc.*	*exc.*	*exc.*	-
TPM	-	*exc.*	*exc.*	*def.**
SMM	*def.*	-	-	*def.*

*Sig. *He* deficiency present only when proportion of SSM was increased to 95% in TPM

Refs.
(1) Cornuet JM, Luikart G (1996) Description and power analysis of two tests for detecting recent population bottlenecks from allele frequency data. Genetics 144: 2001-2014.
(2) Piry S, Luikart G, Cornuet J-M: BOTTLENECK: A Computer Program for Detecting Recent Reductions in the Effective Population Size Using Allele Frequency Data. 1999.
